# Whole-Transcriptome profiling of formalin-fixed, paraffin-embedded renal cell carcinoma by RNA-seq

**DOI:** 10.1186/1471-2164-15-1087

**Published:** 2014-12-11

**Authors:** Ping Li, Andrew Conley, Hao Zhang, Hyung L Kim

**Affiliations:** Department of Surgery, Cedars-Sinai Medical Center, 8635 West Third Street #1070W, Los Angeles, CA 90048 USA; Department of Biomedical Science, Cedars-Sinai Medical Center, Los Angeles, CA USA; Comprehensive Transplant Center, Cedars-Sinai Medical Center, Los Angeles, CA USA

**Keywords:** Formalin-fixed paraffin-embedded (FFPE), qPCR, Renal cell carcinoma (RCC), RNA-seq, Gene expression

## Abstract

**Background:**

Formalin-fixed paraffin-embedded (FFPE) tissue samples are routinely archived in the course of patient care and can be linked to clinical outcomes with long-term follow-up. However, FFPE tissues have degraded RNA which poses challenges for analyzing gene expression. Next-generation sequencing (NGS) is rapidly becoming accepted as an effective tool for measuring gene expressions for research and clinical use. However, the feasibility of NGS has not been firmly established when using FFPE tissue.

**Results:**

We optimized strategies for whole transcriptome sequencing (RNA-seq) using FFPE tissue. Ribosomal RNA (rRNA) was successfully depleted by competitive hybridization using the Ribo-zero™ Kit (Epicentre Biotechnologies), and rRNA sequence content was less than one percent for each library. Gene expression measured by FFPE RNA-seq was compared to two different standards: RNA-seq from fresh frozen (FF) tissue and quantitative PCR (qPCR). Both FF and FFPE tumors were sequenced on an Illumina Genome Analyzer IIX with an average of 10 million reads. The distribution of FPKMs (fragments per kilobase of exon per million fragments mapped) and number of detected genes were similar between FFPE and FF. RNA-seq expressions from FF and FFPE samples from the same renal cell carcinoma (RCC) correlated highly (r = 0.919 for tumor 1 and r = 0.954 for tumor 2). On hierarchical cluster analysis, samples clustered by patient identity rather than method of preservation. TaqMan qPCR of 424 RCC-related genes correlated highly with FFPE RNA-seq expressions (r = 0.775 for FFPE tumor 1, r = 0.803 for FFPE tumor 2). Expression fold changes were considered, to assess biologic relevance of gene expressions. Expression fold changes between FFPE tumors (tumor 1/tumor 2) correlated well when comparing qPCR and RNA-seq (r = 0.890). Expression fold changes between tumors from different risk groups (our high risk RCC/The Cancer Genome Atlas, TCGA, low risk RCC) also correlated well when comparing RNA-seq from FF and FFPE tumors (r = 0.887).

**Conclusions:**

FFPE RNA-seq provides reliable genes expression data, comparable to that obtained from fresh frozen tissue. It represents a useful tool for discovery and validation of biomarkers.

**Electronic supplementary material:**

The online version of this article (doi:10.1186/1471-2164-15-1087) contains supplementary material, which is available to authorized users.

## Background

RNA expression profiling may lead to the discovery of molecular markers for disease diagnosis, assessing prognosis, and targeting with drugs. Quantitative (qPCR) has been the “gold standard” for measuring gene expressions [1, 2] due to its high sensitivity and specificity, reproducibility, and large dynamic range [3–5]. However, next-generation sequencing is rapidly becoming accepted [6] as an effective and more versatile tool for measuring gene expressions for research and clinical use [7–9]. Compared to qPCR, the major advantages of next generation sequencing (NGS) include the ability to analyze a sample’s whole transcriptome in an unbiased way, to discover novel transcripts, and to detect gene fusions, which are common in cancer [10].

The optimal tissue for RNA-seq is fresh frozen (FF) tissue with high quality RNA. Unfortunately, frozen tumors are not widely available because they are costly to collect and maintain. However, formalin-fixed paraffin-embedded (FFPE) tissue samples are routinely archived in the course of patient care and can often be linked to clinical outcomes with long-term follow-up. Unfortunately, FFPE tissues yield relatively low quantities of degraded RNA. A small number of studies have reported using FFPE tissue for whole transcriptome mRNA expression profiling [11–14]. Here, we characterize the performance of RNA-seq on FFPE renal tumors by comparing results to RNA-seq on FF renal tumors and qPCR, which is considered the “gold standard” for measuring gene expression.

In this study, transcriptome-wide RNA-seq was successfully performed on matching FFPE and FF clear cell renal cell carcinomas (ccRCCs). The expression profiles generated from FFPE and FF tumors correlated well. The tumor RNA was also assessed by qPCR using the OpenArray® NT Cycler system, which we’ve previously validated for use with FFPE tumors [15]. RNA quantities measured by RNA-seq and qPCR also correlated well. Expression fold changes between our tumors and tumors from The Cancer Genome Atlas (TCGA) [7] correlated well when expressions from FFPE and FF were compared, suggesting that FFPE RNA-seq can provide biologically meaningful information. We establish the feasibility of using RNA-seq with FFPE tissue and recommend its use in future large-scale RNA-seq studies.

## Results

### Expression levels determined by qPCR

**Figure 1:**
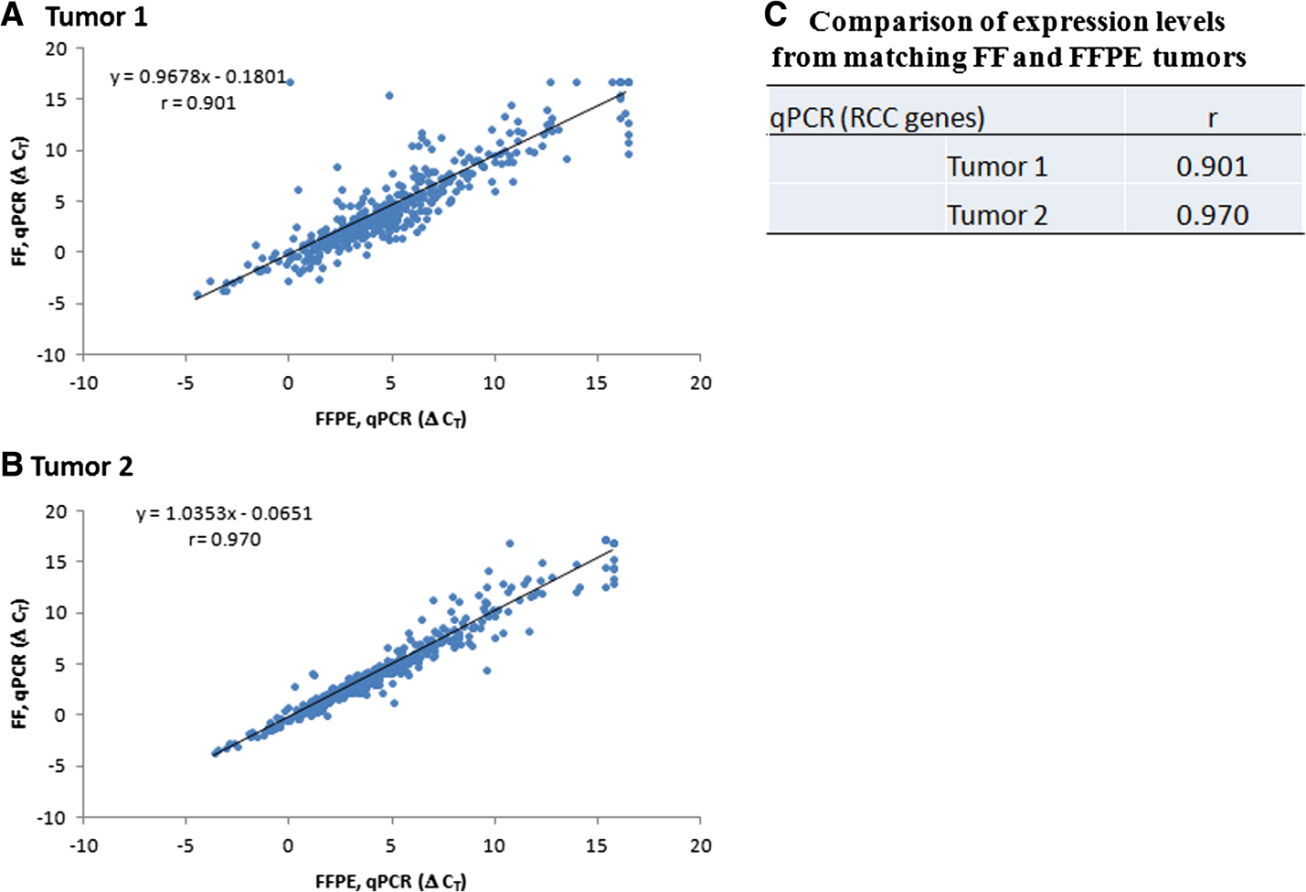
**Correlation of qPCR for matched FF and FFPE tumors. A and B)** The OpenArray® platform was used to perform TaqMan qPCR for RCC genes using matching FF and FFPE tumors. **C)** For qPCR, r (Pearson’s correlation coefficient) was calculated using ΔCT, which is the normalized cycle threshold for each gene. FF, fresh frozen; FFPE, formalin-fixed paraffin-embedded.

TaqMan qPCR is an established assay for determining expression levels using either FF or FFPE tumors. The OpenArray platform uses microfluidics to load nanoliter scale TaqMan qPCR chambers. We recently validated the OpenArray platform for use with FFPE tumors [15]. We conducted a literature review to compile a list of 424 candidate genes relevant to RCC formation, progression, prognosis and response to treatment (referred to as RCC genes). The expression of all RCC genes was quantified using the OpenArray platform for the two matching pairs of FFPE and FF RCCs. The optimal reference genes for normalizing qPCR results (ΔCT) were empirically determined [16]. As expected, the ΔCT for FFPE and FF showed good correlation (Figure 1). This confirms prior reports that qPCR can be used for expression profiling of FFPE tissue. It also indicates that the RNA from our FFPE tumors is of sufficient quality to provide RNA expressions using qPCR, which can serve as a standard for comparing with RNA-seq.

### Library preparation and RNA-seq

**Figure 2:**
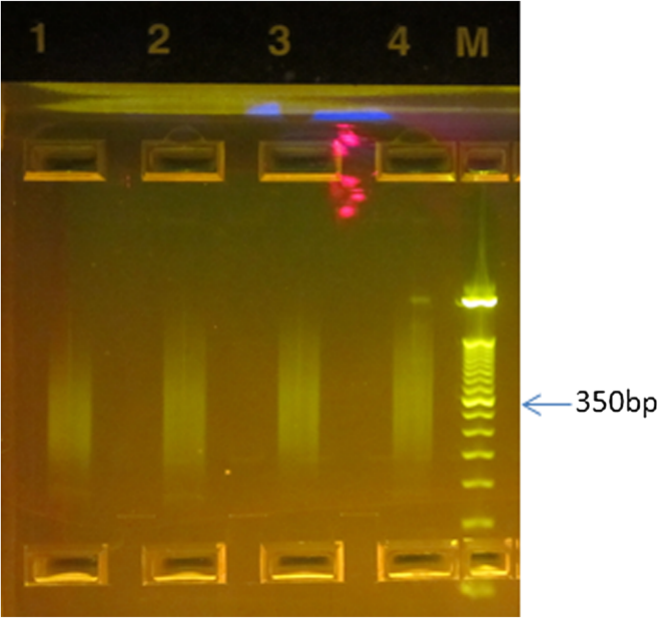
**Library size measurement by E-gel®.** The average size for all the libraries is approximately 300 bp. **Lane 1**: Tumor 1, FFPE; **Lane 2**: Tumor 2, FFPE; **Lane 3**: Tumor 1, FF; **Lane 4**: Tumor 2, FF; **Lane M**: 50 bp marker. FF, fresh frozen; FFPE, formalin-fixed paraffin-embedded.

Total RNA was used to prepare libraries for RNA-seq. Ribosomal RNA (rRNA) constitutes more than 80% of the total tumor RNA. Therefore, to avoid wasting sequencing reactions generating repetitive reads, rRNA was removed by competitive hybridization using the Ribo-Zero™ kit. Total RNA was assessed by Agilent Bioanalyzer before and after Ribo-Zero treatment to show that large peaks corresponding to 18S and 28S rRNA disappeared (Additional file 1: Figure S1). The RNA from FFPE tumor is highly fragmented and the 18S and 28S rRNA peaks were not visible either before or after Ribo-Zero treatment. Sequencing libraries were prepared using ScriptSeq™ v2 RNA-Seq Library Preparation Kit and visualized by E-gel® to confirm fragment sizes of approximately 300 bp (Figure 2).

Average cluster density for the 4 libraries was approximately 800 K/mm2. The average depth of sequencing for each library was 10 million reads. The distribution of FPKMs for all the genes was similar between FFPE and FF (Additional file 2: Figure S2). The number of genes detected by RNA-seq was comparable between FF and FFPE (Additional file 3: Table S1). The distribution of detected genes was also comparable between FF and FFPE (Additional file 4: Figure S3). RNA-seq quality metrics showed that for each library, greater than 70% of the reads were uniquely mapped to the genome with less than 1% being rRNA. The strand specificity was higher than 80%. (Additional file 3: Table S2).

### Comparison of FFPE RNA-seq to established RNA profiling strategies

**Figure 3:**
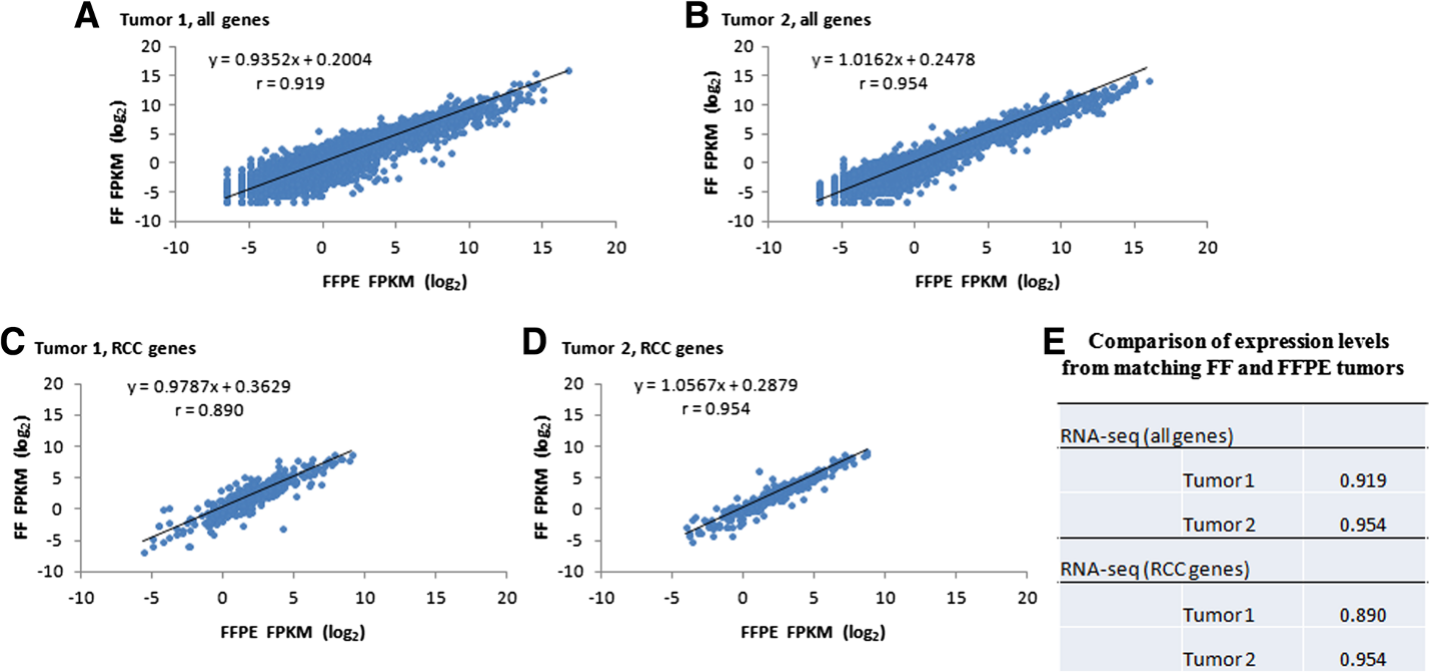
**Correlation of RNA-seq for FF and FFPE tumors. A)** FFPE vs. FF, tumor 1, all RNA-seq genes **B)** FFPE vs. FF, tumor 2, all RNA-seq genes **C)** FFPE vs. FF, tumor 1, RCC genes **D)** FFPE vs. FF, tumor 2, RCC genes **E)** For qPCR, r (Pearson’s correlation coefficient) was calculated using ΔCT. For RNA-seq, r was calculated using the log_2_ FPKM for genes with FPKM > = 0.01. FF, fresh frozen; FFPE, formalin-fixed paraffin-embedded; FPKM, fragments per kilobase of exon per million fragments mapped.

RNA-seq is an established platform for quantifying gene expressions using high quality RNA from FF tissue. To validate expression profiles generated from highly fragment RNA from FFPE tissue, expression profiles were compared from matching pairs of FF and FFPE renal tumors. The expressions were highly correlated (Figure 3), indicating that RNA-seq performs well with FFPE tissue.

**Figure 4:**
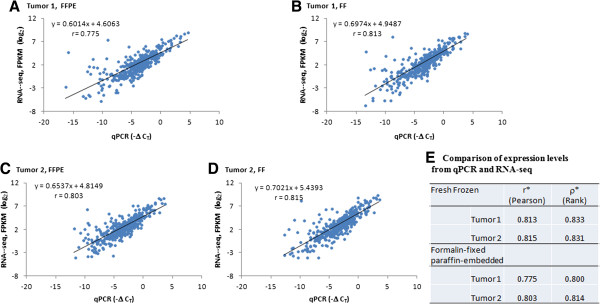
**Correlation of RNA-seq and qPCR for FF and FFPE tumors. A)** Tumor 1, FFPE **B)** Tumor 1, FF **C)** Tumor 2, FFPE **D)** Tumor 2, FF **E)** r (Pearson’s correlation coefficient) and ρ (Spearman’s rank correlation) were calculated using the log_2_ FPKM and -ΔC_T_ for the RCC genes. FF, fresh frozen; FFPE, fresh-frozen paraffin-embedded; FPKM, fragments per kilobase of exon per million fragments mapped.

The gold-standard for quantifying RNA levels from FFPE is TaqMan qPCR, which is used for both research and routine patient care [15, 17]. Our panel of 424 RCC genes were quantified by qPCR using both FF and FFPE tumors and compared with RNA-seq results. The qPCR results are expressed on a log_2_ scale; therefore, FPKM values from RNA-seq were transformed to a log_2_ scale. There was high correlation between expressions determined by qPCR and RNA-seq for both FF and FFPE tumors (Figure 4). Lower ΔCT values indicate higher expression while higher FPKM values indicate higher expression. Figures were plotted using -ΔCT and FPKM. In a similar analysis, rank correlations were considered because the relationship between ΔCT and FPKM may not be linear (Figure 4E, Additional file 5: Figure S4). This analysis confirmed a high correlation between qPCR and RNA-seq for both FF and FFPE tissue.

### FFPE RNA-seq results contain biologically meaningful information

**Figure 5:**
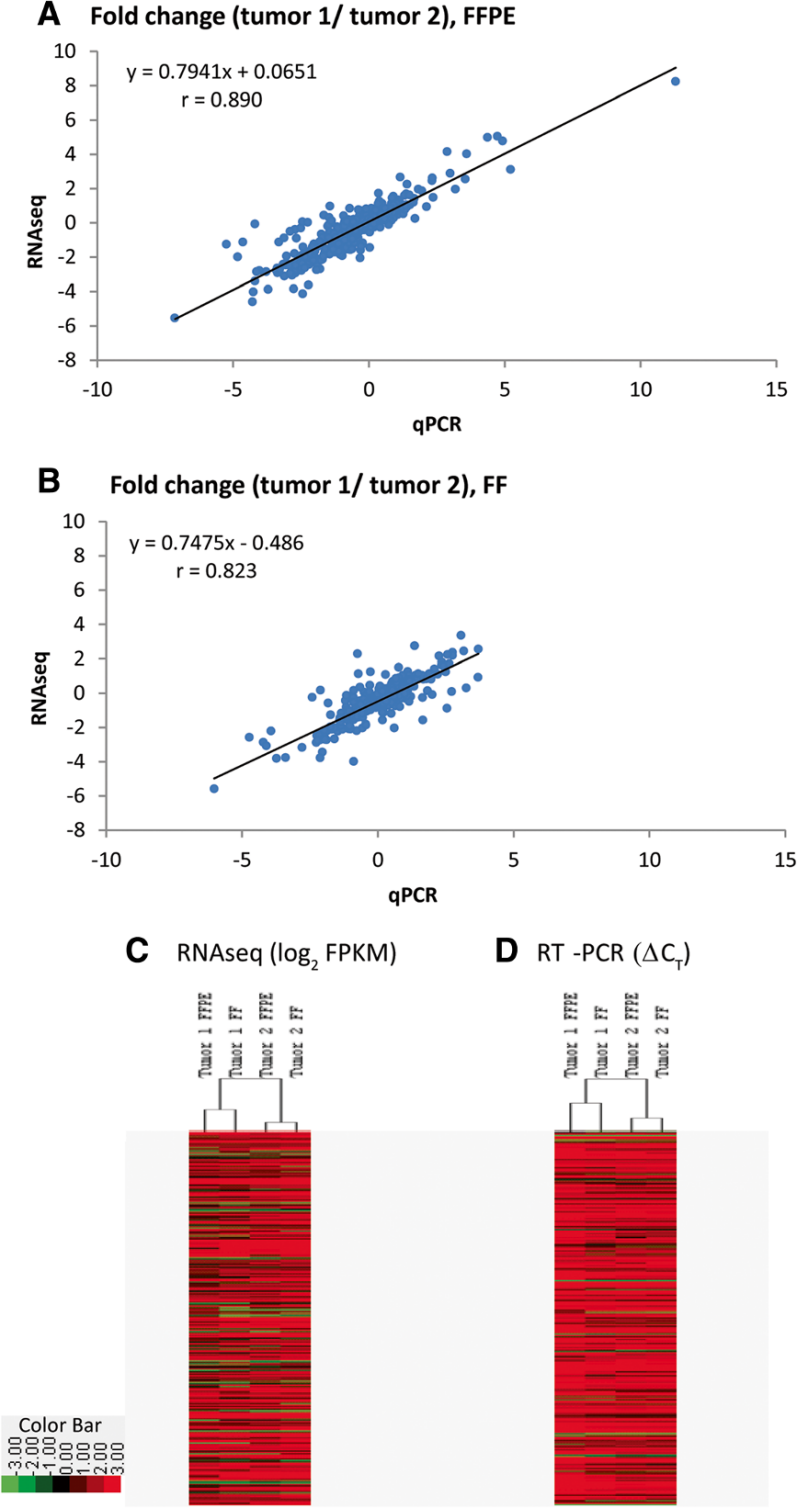
**Potential for biological information. A)** Correlation of expression fold change (between tumor 1 and tumor 2, FFPE) determined using qPCR and RNA-seq. **B)** Same analysis using FF tumors. **C)** Hierarchical clustering based on RCC genes for all four samples after RNA-seq **D)** Same analysis based on qPCR results. FF, fresh frozen; FFPE, fresh-frozen paraffin-embedded.

It is well known that tumor RNAs measured by qPCR or FF RNA-seq reflect diagnostic and prognostic information [7, 18–21]. The tumors used in our study had similar clinical profiles. However they are expected to be molecularly distinct, particularly when the RCC genes are considered. Therefore, fold changes were calculated for each gene comparing tumors from the two patients. If gene expressions from FFPE RNA-seq reflect cancer biology then fold changes should be similar whether the tumor was assessed by RNA-seq or an established platform such as qPCR. This was indeed the case (Figure 5A,B). Possibility of FFPE RNA containing biologic information was further supported by hierarchical cluster analysis performed using RCC genes (Figure 5C,D). The expression profiles clustered based on tumor rather than method used to preserve the tumor, i.e. FF vs FFPE.

**Figure 6:**
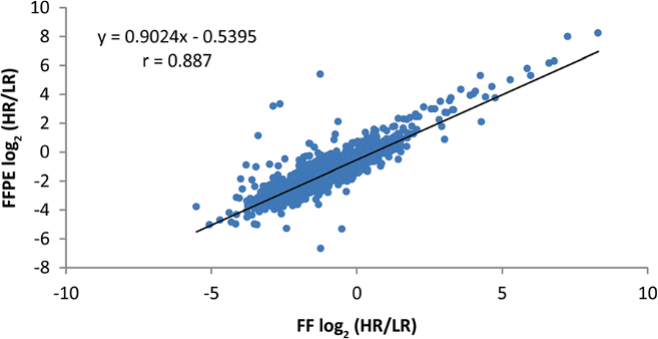
**Correlation of fold changes using FFPE vs FF tumors.** The tumors from our institutional biobank were high risk tumors. To calculate expression fold changes between high risk and low risk tumors, low risk tumors (all FF tumors) were identified from The Cancer Genome Atlas (TCGA). Genes highly expressed by RNA-seq in both our institutional tumors and TCGA tumors were used to calculate fold change. The correlation between fold changes determined from FFPE and FF tumors is shown. The fold change was calculated using log_2_ FPKM. HR, high risk tumors from our institution; LR, low risk tumors from TCGA; FPKM, fragments per kilobase of exon per million fragments mapped.

It is well established that high quality RNA from FF tissue can differentiate between high and low risk tumors. Our tumors were clinically high risk tumors based on stage and grade (Additional file 3: Table S3). They posed high risk for metastatic recurrence even after surgical removal of the primary tumor. To calculate fold change using tumors considered low risk, patients with small, low grade tumors were selected from TCGA. TCGA data was from FF tumors. Expression fold changes were calculated using high risk and low risk tumors. If RNA using FFPE tumors can provide the same biologic information as FF tumors, the fold changes determined using FF or FFPE RCCs should correlate well. This is indeed the case with an r of 0.887 (Figure 6).

## Discussion

The past decade has seen accelerated development of genomic and transcriptomic techniques. A wealth of biologic insights has come from data generated from thousands of tumor samples. Both the cost and turn-around time have dropped dramatically, which in turn, has put this powerful technology within reach of most wet-lab scientists. RNA-seq has become the method of choice for transcriptome profiling. In contrast to microarray and qPCR technologies, RNA-seq can identify novel transcripts, examine all RNA species, and identify alternative splices and mutations.

RCC has been studied using RNA-seq [7, 22]. These studies provide transcriptomic profiles for understanding disease development and prognosis. However, all of these studies used FF tumors as the resource of RNA, and FF tumors are relatively scarce. On the other hand, FFPE samples are widely available because they are routinely stored by all pathology departments. Importantly, FFPE samples often come from patients who have had years of clinical follow-up. Therefore, this clinical annotation can provide the phenotype needed to understand the genomic and molecular data that can now be readily generated.

There are several studies of RNA-seq on FFPE tissue [11–13]. However, all these studies have important limitations. One of the studies used poly-A selection to capture mRNA and exclude ribosomal RNA. This results in the loss of all non-mRNAs and because FFPE RNA is highly degraded, poly-A capture provides only a limited sequence from the 3’ end of the mRNA. Therefore, no information is usually available on alternative splicing and many mutations will be missed [12]. Another study used lungs from two patients who died of influenza to perform FFPE RNA-seq. However, the study profiled viral RNA and not human RNA [13]. A third study from Genomic Health Inc. used FFPE breast cancers; however, there was no comparison with RNA-seq performed with FF breast cancer [11]. This study did establish that the expression of a prognostic signature based on a limited number of genes can be determined using FFPE qPCR or FFPE RNA-seq.

We performed whole-transcriptome RNA-seq on FFPE and FF samples. We demonstrated that gene expressions measured by RNA-seq using FFPE tissue correlate well with FF RNA-seq. Our findings are consistent with a recent report using a similar next-generation sequencing platform as ours to compare RNA from paired FF and FFPE tissue from a variety of tumor types to report a correlation coefficient of 0.90 [14]. This study examined RNA-seq in 38 paired FF and FFPE normal and cancer tissues from bladder, prostate, liver, colon and tonsil. In our study, we examined clear cell RCC. In addition to RNA-seq, we also used qPCR as an established standard for expression profiling and showed good correlation with FFPE RNA-seq. By assessing fold change between patient tumors and performing hierarchical cluster analysis, we showed that FFPE RNA-seq contains potentially meaningful biologic information that can be used for basic discoveries as well as patient care.

A limitation of our study is that we only examined two patient tumors. However, RNA-seq outputs covering the entire transcriptome provide ample data for a robust analysis. Furthermore, use of multiple technology platforms and use of the publically available TCGA results strengthen our conclusion. FFPE tumors, routinely stored by medical centers, are associated with a wealth of clinical data and follow-up information. However, to take advantage of these annotated tissues, RNA-seq needs to be applied to large numbers of archival tissue. Our study, along with other recent, independent reports, establishes the feasibility of RNA-seq for these future studies.

## Conclusion

We demonstrated that gene expressions measured by RNA-seq using FFPE tissue correlate well with two different gold standards: FF RNA-seq and qPCR.

FFPE RNA-seq also provided biological information: The expression profiles from the matched FF and FFPE tissue clustered together. The fold change between high risk FF tumor and TCGA low risk tumors correlated well with the fold change between high risk FFPE tumor and TCGA low risk tumors.

Our study suggests that FFPE RNA-seq can be used for basic discoveries as well as clinical applications.

## Methods

### Patients and tissues

Cedars-Sinai Medical Center Biobank (Los Angeles, CA) provided 4 renal tumors from 2 matching pairs of FFPE and FF ccRCCs. The study was approved by Cedars-Sinai Medical Center’s Institutional Review Board (IRB). For prospectively collected tumor tissue, patients gave written informed consent for data and tissue collection. For FFPE tissue, which already existed in our biobank, IRB provided an exemption for obtaining informed consent. The clinical information associated with each tumor is listed in Additional file 3: Table S1. The FFPE tissues had been stored for about two years at room temperature prior to use in this study.

### RNA Extraction

Our method for RNA extraction has previously been described [15, 16, 23]. Briefly, for FFPE tissue, RNA from six 10-μm sections were extracted using the MasterPure™ RNA Purification Kit (Epicentre Biotechnologies, Madison, WI) with minor modification: 200 ug Proteinase K was added to the Cell Lysis Solution and used for a 3-hr incubation. For FF tissue, RNA was extracted from 100 mg of snap-frozen tissues using TRIzol®. RNA from both FFPE and FF was treated with 20 units DNase I. Complete digestion of genomic DNA was confirmed by TaqMan qPCR for ACTB. The final RNA concentration and purity were measured using a NanoDrop ND-2000 Spectrophotometer (NanoDrop Technologies, Wilmington, DE). The integrity of RNA was measured with the Eukaryote Total RNA Nano Assay of the Agilent 2100 Bioanalyzer (Agilent Technologies, Santa Clara, CA). 200 ng of RNA was loaded for each sample.

### RNA-seq Library preparation and sequencing

Ribosomal RNA was depleted from 4 micrograms of total RNA using the Ribo-Zero™ Magnetic Kit (Epicentre Biotechnologies, Madison, WI) following the manufacturer’s procedure. Sequencing libraries for whole transcriptome analysis were prepared using ScriptSeq™ v2 RNA-Seq Library Preparation Kit (Epicentre Biotechnologies, Madison, WI) following the manufacturer’s procedure. After 3′-terminal tagging, the di-tagged cDNA was purified using MinElute PCR Purification Kit (Qiagen, Valencia, CA). Four 6-base index sequences were used to prepare bar-coded libraries for sample multiplexing (ScriptSeq™ Index PCR Primers (Set 1); Epicentre Biotechnologies, Madison, WI). PCR was carried out through 13 cycles to generate the second strand of cDNA, incorporate barcodes, and amplify libraries. The amplified libraries were size selected by a solid phase reversible immobilization, paramagnetic bead-based process (Agencourt AMPure XP system; Beckman Coulter Genomics, Danvers, MA). The beads: PCR product ratio was 1.8:1.

Libraries were quantified by Qubit® dsDNA HS Assay (Life Technologies, Carlsbad, CA). 15ng libraries were visualized on a 2% E-Gel (Life Technologies, Carlsbad, CA). The four RNA-seq Libraries were then multiplexed in a single lane on a paired end flow cell; clonal amplification was performed using an Illumina cBot (Illumina Inc.; San Diego, CA). Sequencing-by-Synthesis chemistry was performed on an Illumina Genome Analyzer IIX using 35 bp single end sequencing chemistry.

BCL files were converted to FASTQ files using BCLconverter. The FASTQ files were aligned against human genome reference version 19 (or hg19), using TopHat2 (http://ccb.jhu.edu/software/tophat/index.shtml) software package. Transcripts were assembled using Cufflink2 (http://cufflinks.cbcb.umd.edu/). The number of sequenced reads that align to a gene of interest was conventionally called a tag count. To make tag count comparable among samples, we transformed the tag count into FPKM/RPKM (Reads per kilobase per million reads mapped) for normalization, which is widely used in RNA-seq analysis. FPKM value was shown in Additional file 3: Table S4.

To get RNA-seq quality metrics, bedtools utility (http://bedtools.readthedocs.org) was used to overlap TopHat2 mappings with mapping quality of at least 20 and defined exons and introns with a minimum overlap fraction of .8. To reconcile differing exon and intron annotations for a single gene due to alternative splicing, a read was defined as intronic if it was (1) within an intron and (2) overlapped with no exons; reads overlapping any exon were counted as exonic. The bedtools utility was used to determine concordance between the strands of reads mapping to exons and the strands of the exons. To determine the presence of rRNA in the samples, reads were mapped to rRNA human sequences (NR_003287/RNA28S5, NR_003286/ RNA18S5, NR_003285/RNA5-8S5, and NR_023379/ RNA5S17) using the BWA utility (http://bio-bwa.sourceforge.net). Reads which mapped to an rRNA sequence with a mapping quality of at least 20 were considered to be rRNA derived.

### TaqMan qPCR using the open array platform

Reverse transcription (RT) was performed using the High Capacity cDNA Reverse Transcription Kit (Life Technologies, Grand Island, NY) following the manufacturer’s procedure. Each RT reaction contained 150ng of total RNA, 1 μl of 10× RT buffer, 0.5 μl of 25× dNTP mixture, 1 μl of 10× random reverse primers, 1 μl of 10× gene-specific reverse primers (1μM) and 0.5 μl of MultiScribe RT (50 U/μl). The 10 μl reactions were incubated in a Life Technologies Thermocycler for 10 min at 25°C, 2 hours at 37°C and then held at 4°C. The same primers were used for the pre-amplification and the TaqMan qPCR. The 3’end primers used in the PCR were used for the RT.

Preamplification was performed using the TaqMan® PreAmp Master Mix Kit (Life Technologies, Grand Island, NY). Each reaction included 2.5 μl of 2× TaqMan® PreAmp Master Mix, 1.25 μl of 0.09× pooled TaqMan assays (TaqMan primers and probe) and 1.25 μl of cDNA. The reactions were incubated in a Life Technologies Thermocycler for 10 min at 95°C following by 13 cycles of 95°C for 15 seconds and 60°C for 4 min, and then held at 4°C. 0.09× pooled Taqman assays were prepared by combining equal volumes of each 20× Taqman assay (needed for PCR on the Openarray®, 218 assays for each set). Each cDNA was preamplified on two sets of 218 pooled assays.

The two sets of OpenArray plates were custom made. The preamplified products were diluted 1:10; 10 μl of diluted cDNA was mixed with 10 μl of TaqMan Real-time PCR OpenArray Master Mix (Life Technologies, Grand Island, NY). The pre-amplified cDNA samples were dispensed using the Accufill System onto the corresponding OpenArray plate containing 218 gene assays. Twelve cDNA samples were tested simultaneously per plate, with 36 samples per qPCR run on the Openarray® NT Cycler system. Post-acquisition data processing generated fluorescence amplification for each assay, from which cycle threshold (CT) were computed. CT value was shown in Additional file 3: Table S5.

### TCGA RNA-seq data mining and analysis

Clinical information and Level 3 RNA-seq FPKM data were retrieved from the TCGA data portal (https://tcga-data.nci.nih.gov/tcga/). Our tumors were clinically localized tumors that posed high risk for metastasis. To compare these tumors to low risk-RCC using expression fold change, all pT1N0M0, grade 1 or 2 ccRCCs were selected from TCGA (n = 53).

To assess the distribution of FPKMs for all the genes, box plots were generated (Additional file 2: Figure S2). The goal was to identify the most highly expressed genes. The FPKM distribution was different between TCGA data and our data. One of the reasons is that we used different platform for sequencing. TCGA used Hiseq2000. We used GAIIx. Another reason is that we sequenced different cohorts. Based on the distribution of FPKMs, an average FPKM >10 was used to select genes from the TCGA samples. For our samples, an average FPKM >5 was used to select genes. A total of 3,195 overlapping genes common to both datasets were identified and used to calculate expression fold changes between our tumors and TCGA tumors.

### Statistics and bioinformatics

Fold changes in gene expression were calculated: for qPCR results, log_2_ fold change between sample a and b = Δ CT for b - Δ CT for a, and for RNA-seq results, fold change between sample a and b = log2 (FPKM for a/FPKM for b). Complete-linkage Hierarchical clustering was performed using Gene Cluster 3.0 software. The similarity (distance) between gene expression data was defined using Pearson correlation (uncentered). The result was viewed using Java TreeView software.

### Availability of supporting data

The TCGA sequence data and clinical data discussed in this publication are available from the TCGA Data Portal [7].

The supplemental data was available from LabArchive with DOI of 10.6070/H4Q23X6N DOI: 10.6070/H4Q23X6N#doi.

Deep sequencing files have been deposited into Sequence Read Archive under accession number SRP050335.

## Electronic supplementary material

Additional file 1: Figure S1: RNA integrity test before and after Ribo-zero treatment. 200ng of RNA was measured with the Eukaryote Total RNA Nano Assay of the Agilent 2100 Bioanalyzer. FF, fresh frozen; FFPE: formalin-fixed paraffin-embedded; Before, before ribo-zero treatment; After, after ribo-zero treatment. (TIFF 1 MB)

Additional file 2: Figure S2: Box Graph for RNA-seq. Distribution of FPKMs determined from RNA-seq. Horizontal line inside the box is the median expression. The box contains expressions between the 25^th^ and 75^th^ percentile. TCGA, The Cancer Genome Atlas RCC; CS FF, Cedars Sinai fresh frozen RCC; CS FFPE, Cedars Sinai formalin-fixed paraffin-embedded RCC. (TIFF 544 KB)

Additional file 3: **Supplemental Tables.** (XLSX 973 KB)

Additional file 4: Figure S3: Distribution of detected genes for RNA-seq. Transcript counts are plotted against Log2 (FPKM) for genes determined from FF and FFPE RNA-seq. FF, fresh frozen; FFPE, fresh-frozen paraffin-embedded; FPKM, fragments per kilobase of exon per million fragments mapped. (TIFF 578 KB)

Additional file 5: Figure S4: Rank correlation of RNA-seq and qPCR for matched FF and FFPE tumors. A) Tumor 1, FFPE B) Tumor 1, FF C) Tumor 2, FFPE D) Tumor 2, FF. FF, fresh frozen; FFPE, fresh-frozen paraffin-embedded; FPKM, fragments per kilobase of exon per million fragments mapped. (TIFF 1 MB)

## References

[1] Mackay IM, Arden KE, Nitsche A (2002). Real-time PCR in virology. Nucleic Acids Res.

[2] van’t Veer LJ, Bernards R (2008). Enabling personalized cancer medicine through analysis of gene-expression patterns. Nature.

[3] Arya M, Shergill IS, Williamson M, Gommersall L, Arya N, Patel HR (2005). Basic principles of real-time quantitative PCR. Expert Rev Mol Diagn.

[4] Wilhelm J, Pingoud A (2003). Real-time polymerase chain reaction. Chembiochem.

[5] Wong ML, Medrano JF (2005). Real-time PCR for mRNA quantitation. Biotechniques.

[6] Wang Z, Gerstein M, Snyder M (2009). RNA-Seq: a revolutionary tool for transcriptomics. Nat Rev Genet.

[7] Cancer Genome Atlas Research Network (2013). Comprehensive molecular characterization of clear cell renal cell carcinoma. Nature.

[8] Tuch BB, Laborde RR, Xu X, Gu J, Chung CB, Monighetti CK, Stanley SJ, Olsen KD, Kasperbauer JL, Moore EJ, Broomer AJ, Tan R, Brzoska PM, Muller MW, Siddiqui AS, Asmann YW, Sun Y, Kuersten S, Barker MA, De La Vega FM, Smith DI (2010). Tumor transcriptome sequencing reveals allelic expression imbalances associated with copy number alterations. PLoS One.

[9] Guo G, Sun X, Chen C, Wu S, Huang P, Li Z, Dean M, Huang Y, Jia W, Zhou Q, Tang A, Yang Z, Li X, Song P, Zhao X, Ye R, Zhang S, Lin Z, Qi M, Wan S, Xie L, Fan F, Nickerson ML, Zou X, Hu X, Xing L, Lv Z, Mei H, Gao S, Liang C (2013). Whole-genome and whole-exome sequencing of bladder cancer identifies frequent alterations in genes involved in sister chromatid cohesion and segregation. Nat Genet.

[10] Maher CA, Kumar-Sinha C, Cao X, Kalyana-Sundaram S, Han B, Jing X, Sam L, Barrette T, Palanisamy N, Chinnaiyan AM (2009). Transcriptome sequencing to detect gene fusions in cancer. Nature.

[11] Sinicropi D, Qu K, Collin F, Crager M, Liu ML, Pelham RJ, Pho M, Dei Rossi A, Jeong J, Scott A, Ambannavar R, Zheng C, Mena R, Esteban J, Stephans J, Morlan J, Baker J (2012). Whole transcriptome RNA-Seq analysis of breast cancer recurrence risk using formalin-fixed paraffin-embedded tumor tissue. PLoS One.

[12] Beck AH, Weng Z, Witten DM, Zhu S, Foley JW, Lacroute P, Smith CL, Tibshirani R, van de Rijn M, Sidow A, West RB (2010). 3’-end sequencing for expression quantification (3SEQ) from archival tumor samples. PLoS One.

[13] Xiao YL, Kash JC, Beres SB, Sheng ZM, Musser JM, Taubenberger JK (2013). High-throughput RNA sequencing of a formalin-fixed, paraffin-embedded autopsy lung tissue sample from the 1918 influenza pandemic. J Pathol.

[14] Hedegaard J, Thorsen K, Lund MK, Hein AM, Hamilton-Dutoit SJ, Vang S, Nordentoft I, Birkenkamp-Demtröder K, Kruhøffer M, Hager H, Knudsen B, Andersen CL, Sørensen KD, Pedersen JS, Ørntoft TF, Dyrskjøt L (2014). Next-generation sequencing of RNA and DNA isolated from paired fresh-frozen and formalin-fixed paraffin-embedded samples of human cancer and normal tissue. PLoS One.

[15] Li P, Grigorenko E, Funari V, Enright E, Zhang H, Kim HL (2013). Evaluation of a high-throughput, microfluidics platform for performing TaqMan™ qPCR using formalin-fixed paraffin-embedded tumors. Bioanalysis.

[16] Glenn ST, Jones CA, Liang P, Kaushik D, Gross KW, Kim HL (2007). Expression profiling of archival renal tumors by quantitative PCR to validate prognostic markers. Biotechniques.

[17] Thompson E, Burt AD, Barker CE, Kirby JA, Brain JG (2013). Development of a robust protocol for gene expression analysis using formalin-fixed, paraffin-embedded liver transplant biopsy specimens. J Clin Pathol.

[18] Takahashi M, Rhodes DR, Furge KA, Kanayama H, Kagawa S, Haab BB, Teh BT (2001). Gene expression profiling of clear cell renal cell carcinoma: gene identification and prognostic classification. Proc Natl Acad Sci U S A.

[19] Zhao H, Ljungberg B, Grankvist K, Rasmuson T, Tibshirani R, Brooks JD (2006). Gene expression profiling predicts survival in conventional renal cell carcinoma. PLoS Med.

[20] Vasselli JR, Shih JH, Iyengar SR, Maranchie J, Riss J, Worrell R, Torres-Cabala C, Tabios R, Mariotti A, Stearman R, Merino M, Walther MM, Simon R, Klausner RD, Linehan WM (2003). Predicting survival in patients with metastatic kidney cancer by gene-expression profiling in the primary tumor. Proc Natl Acad Sci U S A.

[21] Brannon AR, Reddy A, Seiler M, Arreola A, Moore DT, Pruthi RS, Wallen EM, Nielsen ME, Liu H, Nathanson KL, Ljungberg B, Zhao H, Brooks JD, Ganesan S, Bhanot G, Rathmell WK (2010). Molecular stratification of clear cell renal cell carcinoma by consensus clustering reveals distinct subtypes and survival patterns. Genes Cancer.

[22] Sato Y, Yoshizato T, Shiraishi Y, Maekawa S, Okuno Y, Kamura T, Shimamura T, Sato-Otsubo A, Nagae G, Suzuki H, Nagata Y, Yoshida K, Kon A, Suzuki Y, Chiba K, Tanaka H, Niida A, Fujimoto A, Tsunoda T, Morikawa T, Maeda D, Kume H, Sugano S, Fukayama M, Aburatani H, Sanada M, Miyano S, Homma Y, Ogawa S (2013). Integrated molecular analysis of clear-cell renal cell carcinoma. Nat Genet.

[23] Glenn ST, Head KL, Teh BT, Gross KW, Kim HL (2010). Maximizing RNA yield from archival renal tumors and optimizing gene expression analysis. J Biomol Screen.

