# Supplementary figures and images for: Whole-Transcriptome profiling of formalin-fixed, paraffin-embedded renal cell carcinoma by RNA-seq
Source: BMC Genomics. 2014 Dec 11;15(1):1087. doi: 10.1186/1471-2164-15-1087 (PMC4298956; doi:10.1186/1471-2164-15-1087)

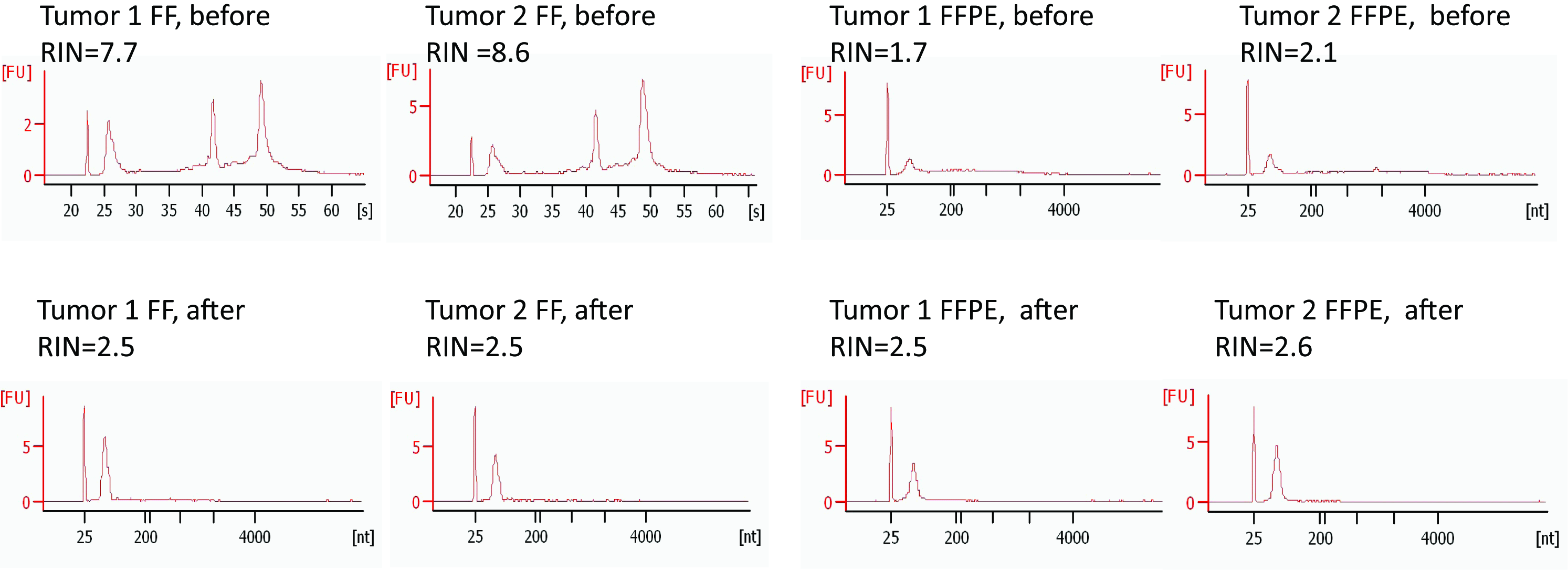

Supplement: Supplementary file 1 — Additional file 1: Figure S1: RNA integrity test before and after Ribo-zero treatment. 200ng of RNA was measured with the Eukaryote Total RNA Nano Assay of the Agilent 2100 Bioanalyzer. FF, fresh frozen; FFPE: formalin-fixed paraffin-embedded; Before, before ribo-zero treatment; After, after ribo-zero treatment. (TIFF 1 MB) [file 12864_2014_6897_MOESM1_ESM.tiff]

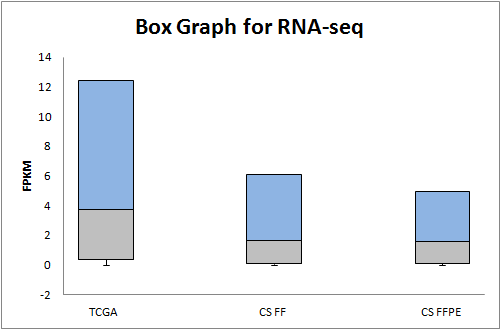

Supplement: Supplementary file 2 — Additional file 2: Figure S2: Box Graph for RNA-seq. Distribution of FPKMs determined from RNA-seq. Horizontal line inside the box is the median expression. The box contains expressions between the 25th and 75th percentile. TCGA, The Cancer Genome Atlas RCC; CS FF, Cedars Sinai fresh frozen RCC; CS FFPE, Cedars Sinai formalin-fixed paraffin-embedded RCC. (TIFF 544 KB) [file 12864_2014_6897_MOESM2_ESM.tiff]

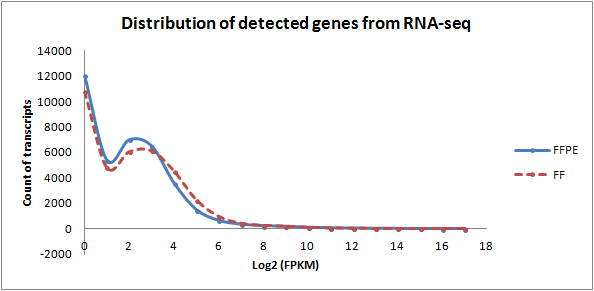

Supplement: Supplementary file 4 — Additional file 4: Figure S3: Distribution of detected genes for RNA-seq. Transcript counts are plotted against Log2 (FPKM) for genes determined from FF and FFPE RNA-seq. FF, fresh frozen; FFPE, fresh-frozen paraffin-embedded; FPKM, fragments per kilobase of exon per million fragments mapped. (TIFF 578 KB) [file 12864_2014_6897_MOESM4_ESM.tiff]

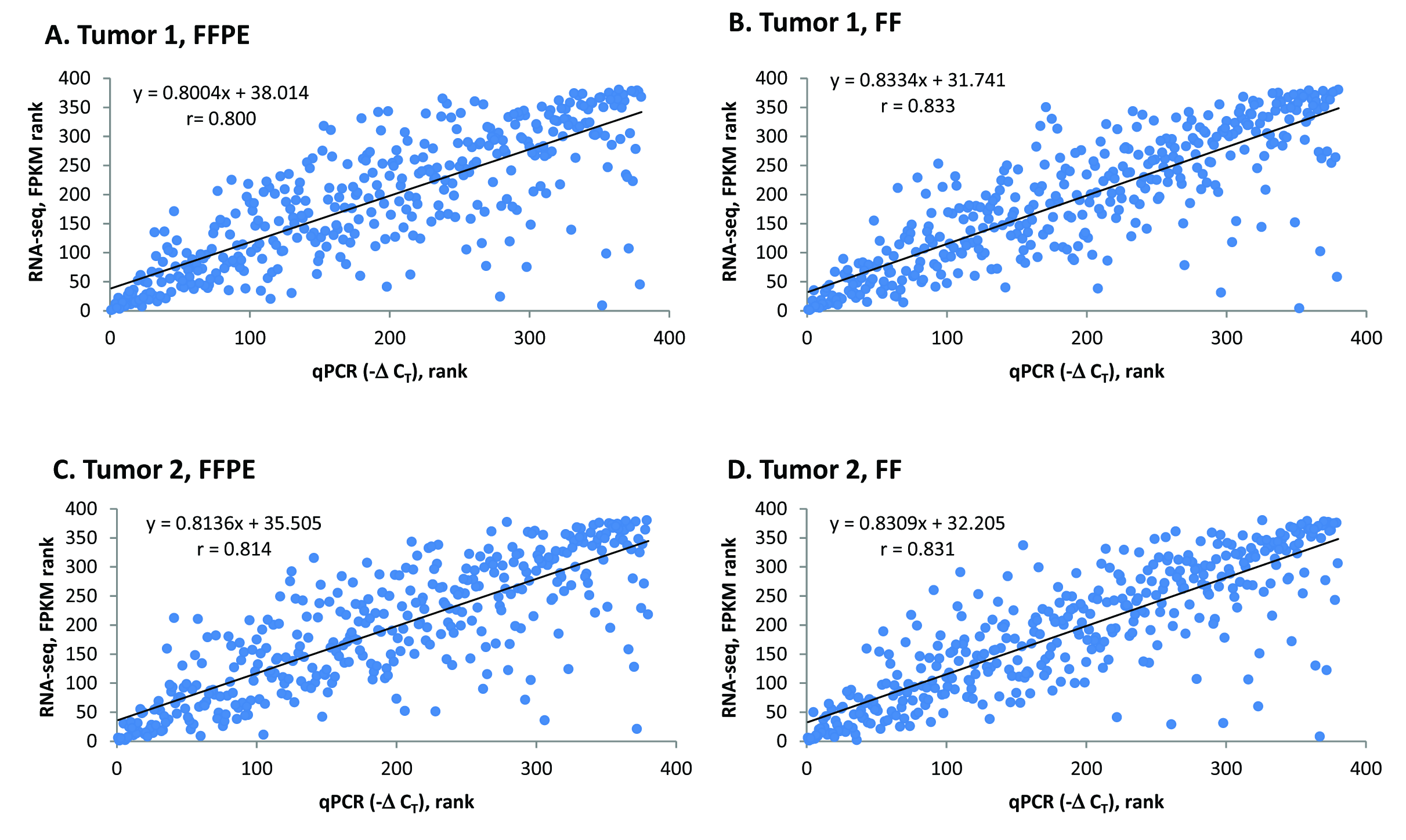

Supplement: Supplementary file 5 — Additional file 5: Figure S4: Rank correlation of RNA-seq and qPCR for matched FF and FFPE tumors. A) Tumor 1, FFPE B) Tumor 1, FF C) Tumor 2, FFPE D) Tumor 2, FF. FF, fresh frozen; FFPE, fresh-frozen paraffin-embedded; FPKM, fragments per kilobase of exon per million fragments mapped. (TIFF 1 MB) [file 12864_2014_6897_MOESM5_ESM.tiff]
